# A New RING Finger Protein, *PLANT ARCHITECTURE* and *GRAIN NUMBER* 1, Affects Plant Architecture and Grain Yield in Rice

**DOI:** 10.3390/ijms23020824

**Published:** 2022-01-13

**Authors:** Peiwen Yan, Yu Zhu, Ying Wang, Fuying Ma, Dengyong Lan, Fuan Niu, Shiqing Dong, Xinwei Zhang, Jian Hu, Siwen Liu, Tao Guo, Xiaoyun Xin, Shiyong Zhang, Jinshui Yang, Liming Cao, Xiaojin Luo

**Affiliations:** 1 State Key Laboratory of Genetic Engineering and MOE Engineering Research Center of Gene Technology, School of Life Sciences, Fudan University, Shanghai 200438, China; 19210700063@fudan.edu.cn (P.Y.); 18210700055@fudan.edu.cn (Y.Z.); wangying09@fudan.edu.cn (Y.W.); 20110700053@fudan.edu.cn (F.M.); 20210700053@fudan.edu.cn (D.L.); niufuan224@126.com (F.N.); 18110700036@fudan.edu.cn (S.D.); 21210700121@m.fudan.edu.cn (X.Z.); 21210700028@m.fudan.edu.cn (J.H.); 13548788475@163.com (S.L.); xinxy@fudan.edu.cn (X.X.); jsyang@fudan.edu.cn (J.Y.); 2 MOE Key Laboratory of Crop Physiology, Ecology and Genetic Breeding College of Agronomy, Jiangxi Agricultural University, Nanchang 330045, China; 3 Institute of Crop Breeding and Cultivation, Shanghai Academy of Agricultural Sciences, Shanghai 201403, China; 4 Institute of Wetland Agriculture and Ecology, Shandong Academy of Agricultural Sciences/Shandong Rice Engineering Technology Research Center, Jinan 250100, China; guotaorice@163.com (T.G.); 13305371992@163.com (S.Z.)

**Keywords:** RING finger protein, plant architecture, grain yield, cytokinin

## Abstract

Developing methods for increasing the biomass and improving the plant architecture is important for crop improvement. We herein describe a gene belonging to the RING_Ubox (RING (Really Interesting New Gene) finger domain and U-box domain) superfamily, *PLANT ARCHITECTURE and* *GRAIN NUMBER 1* (*PAGN1*), which regulates the number of grains per panicle, the plant height, and the number of tillers. We used the CRISPR/Cas9 system to introduce loss-of-function mutations to *OsPAGN1*. Compared with the control plants, the resulting *pagn1* mutant plants had a higher grain yield because of increases in the plant height and in the number of tillers and grains per panicle. Thus, *OsPAGN1* may be useful for the genetic improvement of plant architecture and yield. An examination of evolutionary relationships revealed that *OsPAGN1* is highly conserved in rice. We demonstrated that OsPAGN1 can interact directly with OsCNR10 (CELL NUMBER REGULATOR10), which negatively regulates the number of rice grains per panicle. A transcriptome analysis indicated that silencing *OsPAGN1* affects the levels of active cytokinins in rice. Therefore, our findings have clarified the *OsPAGN1* functions related to rice growth and grain development.

## 1. Introduction

Rice, which is an important food crop cultivated worldwide, is the main food source for more than half of the global population. Because of rapid population growth and a sharp decrease in agricultural land area, there is an urgent need for higher yielding rice varieties. Increasing the biomass is the main method for optimizing the rice grain yield [1]. Regarding rice improvement-related research, one of the key target traits is plant architecture, including plant height, tiller number, and panicle morphology, all of which influence the final biomass and grain yield. The genetic mechanism controlling the rice grain yield has been widely studied, and many quantitative trait loci (QTLs) related to grain number have been identified. The *Ideal Plant Architecture 1* (*IPA1*) gene encodes SQUAMOSA PROMOTER-BINDING PROTEIN-LIKE 14 (OsSPL14). Compared with control plants, *OsSPL14*^ipa1^ plants are reportedly taller and have denser panicles and fewer tillers (with few unproductive tillers). A previous study revealed that *OsSPL14* expression is regulated by OsmiR156 via direct transcriptional cleavage and translational repression [2]. The *Grain Number, Plant Height and Heading Date7* (*Ghd7*) gene has a key role affecting photoperiodic flowering. An earlier investigation demonstrated that up-regulated *Ghd7* expression can positively affect rice plant height and grain production [3]. The protein encoded by *MONOCULM 1* (*MOC1*) positively affects tillering by controlling axillary meristem initiation and tiller bud formation [4]. A recent study confirmed that MOC1 can interact with the DELLA protein SLENDER RICE 1 (SLR1), which is a key component of the gibberellin signaling pathway that modulates tiller development [5]. A gain-of-function mutation to *OsDEP1* (*DENSE AND ERECT PANICLE1*) results in an increase in the number of grains per panicle and a consequent increase in grain yield. Recent research indicated that DEP1, which is a Gγ protein, antagonistically regulates grain size with two other Gγ proteins, GS3 and GGC2, through the G protein signaling pathway [6].

Ubiquitination is involved in the regulation of diverse cellular processes [7]. The ubiquitin/proteosome pathway (i.e., UPS pathway) mainly consists of the following components: ubiquitin-activating enzyme (E1), ubiquitin-conjugating enzyme (E2), ubiquitin ligase (E3), and 26S proteasome. In this pathway, E2 either transfers ubiquitin directly to E3 or binds to E3 and facilitates the transfer of ubiquitin to the substrate, after which the 26S proteasome degrades the polyubiquitinated proteins [8,9]. Because E3 is responsible for specifically recognizing and binding substrates, it has been the most widely studied component of the UPS pathway. Many recent studies have elucidated the regulatory effects of the UPS pathway on rice yield. TRANSPORT INHIBITOR RESISTANT1/AUXIN SIGNALING F-BOX (TIR1/AFB), which is a ubiquitin ligase, is an auxin receptor that mediates the degradation of Aux/IAA proteins [10,11,12]. Mutations to *OsTIR1* and *OsAFB2* can significantly alter many rice agronomic traits, including plant height, tillering, and the number of filled grains per panicle [13]. In rice, *GRAIN WIDTH 2* (*GW2*) regulates the grain width and weight [14]. Researchers recently reported that GW2 may be an E3 ubiquitin ligase that ubiquitinates expansin-like 1 (EXPLA1) to promote its degradation to control grain size [15].

Many proteins belonging to the RING_Ubox (RING (Really Interesting New Gene) finger domain and U-box domain) superfamily are E3 ubiquitin ligases [16]. In plants, RING finger proteins are involved in a series of physiological processes related to the photoperiod as well as leaf and root development [17]. Several RING finger proteins in rice have been identified and characterized. CONSTITUTIVELY PHOTOMORPHOGENIC 1 (COP1) was identified as a central regulator of photomorphogenic development in plants [18]. The COP1 ortholog (PPS) in rice regulates the vegetative phase transition and the flowering time [19]. *POLLEN TUBE BLOCKED 1* (*PTB1*) encodes a RING-type E3 ligase and positively regulates the rice panicle seed setting rate by promoting pollen tube growth [20]. Another RING finger E3 ligase, IPA1-INTERACTING PROTEIN 1 (IPI1), promotes the degradation of IPA1 in panicles to modify the plant architecture [21].

Despite the successful cloning and characterization of the above-mentioned genes, the molecular mechanism and regulatory network underlying rice plant architecture and grain yield have not been thoroughly characterized. The CRISPR/Cas9 (clustered regularly interspaced short palindromic repeats/CRISPR-associated Cas9) system has been widely used for editing plant genomes because of its simplicity and high efficiency. The CRISPR/Cas9 system can be used for functional genomics research and for improving crop varieties [22]. In this study, we analyzed the rice-specific gene *PLANT ARCHITECTURE and GRAIN NUMBER 1* (*OsPAGN1*), which encodes an uncharacterized RING finger protein. Silencing *OsPAGN1* expression resulted in increases in the plant height, the number of tillers, and the number of grains per plant. An examination of gene expression (i.e., RNA-seq analysis) suggested that *OsPAGN1* might affect the expression of cytokinin-related genes, thereby regulating rice growth and development. Furthermore, OsPAGN1 was revealed to interact with CELL NUMBER REGULATOR10 (OsCNR10), which negatively affected the rice grain yield.

## 2. Results

### 2.1. OsPAGN1 Is Involved in the Regulation of the Rice Plant Architecture and Grain Yield

To explore the roles of the RING finger family genes affecting rice growth and development, we first analyzed the expression levels of all RING family genes in different tissues and stages by searching the Rice Genome Annotation Project database (http://rice.plantbiology.msu.edu/ (accessed on 10 December 2021)). To investigate the potential effects of rice-specific RING finger proteins on rice grains, we selected RING finger genes lacking homologs in other species and eliminated genes with low expression levels in seedlings and panicles. Of the 324 RING finger family genes, five were retained (Table S1). In this study, we functionally characterized only *LOC_Os07g23970*.

The *LOC_Os07g23970* gene likely encodes an E3 ubiquitin ligase comprising 146 amino acids, including a RING-H2 finger domain (Figure S1). We identified homologous proteins by conducting BLAST searches of the UniProt database (https://www.uniprot.org/ (accessed on 10 December 2021)). Homologs with the conserved domain of LOC_Os07g23970.1 (100–143 amino acids) were detected in several plant species (Table S2), including rice, banana (*Musa balbisiana*), orchid (*Apostasia shenzhenica*), pineapple (*Ananas comosus*), and major grain crops, including maize (*Zea mays*), proso millet (*Panicum miliaceum*), sorghum (*Sorghum bicolor*), and foxtail millet (*Setaria italica*). Homologs comprising the full-length of LOC_Os07g23970.1 were identified only in rice, including *Oryza sativa* subsp. *japonica*, *O. sativa* subsp. *indica*, and wild rice species (Table S3). All of these homologs were similar to LOC_Os07g23970.1 (above 73.6% sequence identity), indicating that *LOC_Os07g23970* is highly conserved in rice.

**Figure 1:**
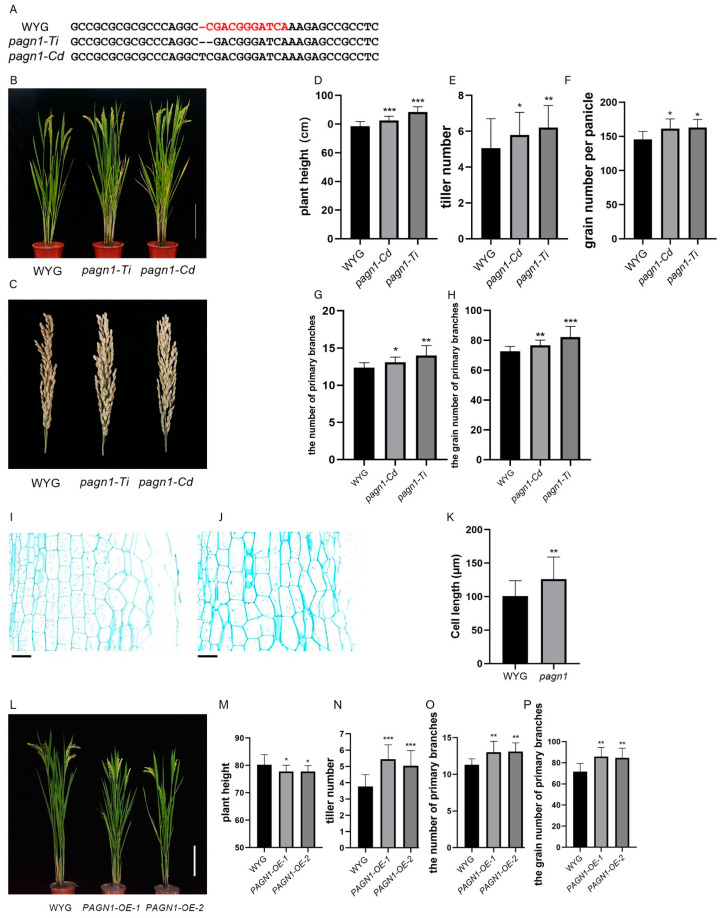
Phenotypes of *OsPAGN1* transgenic lines. (**A**) Target site in the *OsPAGN1* sequence in the *OsPAGN1* knockout lines *pagn1-Ti* and *pagn1-Cd*. (**B**) Plant architecture and (**C**) the panicles of the wild-type (Wuyugeng) and *OsPAGN1* knockout lines. (**D**–**H**) Plant height, tiller number, grain number per panicle, number of primary branches, and grain number for the primary branches of the wild-type and *OsPAGN1* knockout lines. (**I**–**K**) Stem cell length of the wild-type and *OsPAGN1* knockout lines. Bar = 100 μm. (**L**) Plant architecture of the wild-type and *OsPAGN1*-overexpressing lines. (**M**–**P**) Plant height, tiller number, number of primary branches, and grain number for the primary branches of the wild-type and *OsPAGN1*-overexpressing lines. The data are the mean ± SEM. * *p* < 0.05, ** *p* < 0.01, *** *p* < 0.001. (Student’s *t* test).

We mutated *LOC_Os07g23970* in the Wuyugeng rice background using the CRISPR/Cas9 system. The resulting two transgenic lines had an enhanced plant architecture and produced more grains than the control plants. Accordingly, we designated *LOC_Os07g23970* as *PLANT ARCHITECTURE and GRAIN NUMBER 1* (*PAGN1*) and the two knockout transgenic lines as *pagn1-Ti* and *pagn1-Cd* (Figure 1A). Compared with the wild-type plants, the *OsPAGN1* knockout lines were significantly taller and had more tillers per plant and grains per panicle (Figure 1B–F). The increase in plant height was mainly because of stem cell elongation (Figure 1I–K). The increase in the number of grains per panicle was mainly attributed to significant increases in the number of primary branches and the number of grains from the primary branches (Figure 1G,H). We also generated two *OsPAGN1*-overexpressing transgenic lines, which we named *PAGN1-OE-1* and *PAGN1-OE-2*. As expected, the *OsPAGN1*-overexpressing plants were shorter than Wuyugeng, but they produced more tillers, primary branches, and grains from the primary branches (Figure 1L–P). These findings imply *OsPAGN1* is involved in the regulation of the rice plant height and grain yield.

### 2.2. OsPAGN1 Interacts with OsCNR10, Which Controls the Rice Grain Yield

**Figure 2:**
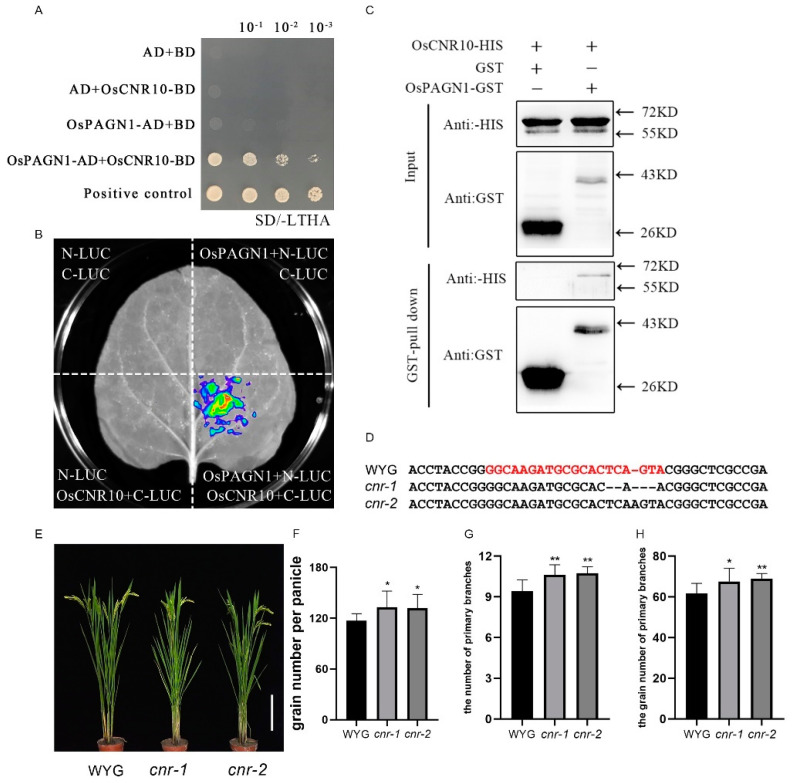
Interaction between OsPAGN1 and OsCNR10. (**A**) Yeast two-hybrid assay of OsPAGN1 and OsCNR10. More specifically, OsCNR10 was fused with the DNA-binding domain (BD) of GAL4, whereas OsPAGN1 was fused with the activation domain (AD) of GAL4. Yeast cells transformed with the recombinant plasmids were grown on the synthetic defined medium lacking Leu, Trp, His, and Ade (SD−LTHA). (**B**) Split-luciferase assay. OsPAGN1 and OsCNR10 were fused with the N-terminal (nLUC) and C-terminal (cLUC) portions of firefly luciferase (LUC), respectively. Different combinations of constructs were inserted into tobacco leaves and then the chemiluminescence was detected after adding the substrate luciferin. (**C**) Pull-down assay. OsPAGN1 and OsCNR10 were fused with the GST and HIS tags, respectively. After a co-incubation with both proteins, the proteins were immunoprecipitated using glutathione resin and analyzed using anti-HIS and anti-GST antibodies. Phenotypes of the *OsCNR10* knockout lines. (**D**) Target site in the *OsCNR10* sequence in the *OsCNR10* knockout lines *cnr-1* and *cnr-2* (**E**) Plant architecture of the wild-type (Wuyugeng) and *OsCNR10* knockout lines. (**F**–**H**) Grain number per panicle, number of primary branches, and grain number for the primary branches of the wild-type and *OsPAGN1* knockout lines. The data are the mean ± SEM. * *p* < 0.05, ** *p* < 0.01. (Student’s *t* test).

We screened for OsPAGN1-interacting proteins via a yeast two-hybrid assay with OsPAGN1 as the bait. A total of 19 candidate proteins were detected, including OsCNR10. The interaction between OsPAGN1 and OsCNR10 was verified by a yeast two-hybrid and one-to-one verification assay (Figure 2A). To further confirm the interaction, we performed a luciferase complementation assay involving *N. benthamiana* leaves transiently expressing the OsPAGN1-nLUC and OsCNR10-cLUC fusion proteins. The negative control leaves contained nLUC and cLUC, nLUC and OsCNR10-cLUC, or OsPAGN1-nLUC and cLUC. Strong fluorescence was detected in the leaves expressing OsPAGN1-nLUC and OsCNR10-cLUC, whereas the signal was undetectable in the negative controls (Figure 2B). These results indicate that OsPAGN1 interacts with OsCNR10 in *N. benthamiana*.

We also performed a GST pull-down assay. More specifically, GST-OsPAGN1 and His-OsCNR10 expressed in *E. coli* were purified and then His-OsCNR10 was incubated with GST-OsPAGN1 or GST. Proteins were precipitated using GST beads and analyzed in an immunoblot involving anti-GST and anti-His antibodies. The His-OsCNR10 fusion protein was detected when it was incubated with GST-OsPAGN1, but not when it was incubated with GST (Figure 2C), indicating OsPAGN1 interacts with OsCNR10.

To investigate whether *OsCNR10* affects rice plant architecture, we silenced *OsCNR10* in Wuyugeng (Figure 2D). The lack of *OsCNR10* expression increased grain production, which is consistent with the results of a previous study on maize [23]. Similar to the *OsPAGN1* knockout lines, the increase in the number of grains was because of increases in the number of primary branches and the number of grains from the primary branches (Figure 2E–H).

### 2.3. OsPAGN1 Expression Patterns

**Figure 3:**
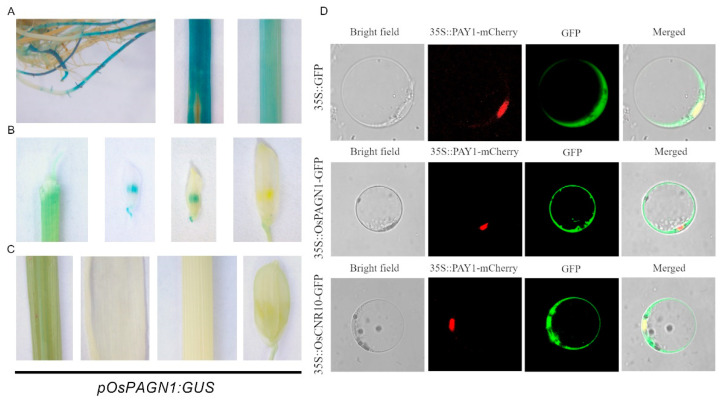
*OsPAGN1* expression patterns. (**A**–**C**) *OsPAGN1* promoter activities in rice tissues as determined by promoter–GUS assays. An approximately 2-kb sequence upstream of the ATG codon of *OsPAGN1* was inserted upstream of the β-glucuronidase (GUS) reporter gene. (**A**) Representative roots, leaf, and stem at the three-leaf stage. (**B**) Spikelet of panicles 0.5, 2, 6, or 10 cm long. (**C**) Representative leaf, shoot, stem, and spikelet at the booting stage. (**D**) Subcellular localization of OsPAGN1 and OsCNR10 in rice protoplasts.

To characterize the *OsPAGN1* expression patterns in rice tissues, we constructed a transgenic line containing the GUS reporter. At the three-leaf stage, GUS expression was observed in the roots, leaves, and stems (Figure 3A). At the booting stage, the *OsPAGN1* promoter was active in the anthers when the panicles were shorter than 6 cm, but there was no detectable GUS activity in spikelets when the panicles were longer than 10 cm (Figure 3B) or in the other examined tissues (Figure 3C). Accordingly, *OsPAGN1* may be associated with early grain development and seedling development.

The subcellular localization of OsPAGN1 was investigated using the *Pro35S::OsPAGN1-GFP* and *Pro35S::PAY1-RFP* constructs, the latter of which was used as a nuclear localization marker in transformed rice protoplasts [24]. We detected the fluorescence of the OsPAGN1-GFP fusion protein in the cytoplasm, but not in the nucleus, reflecting the cytoplasmic localization of OsPAGN1 (Figure 3D). Regarding OsCNR10, in addition to being detected in the cytoplasm along with OsPAGN1, it was also present in the nucleus.

### 2.4. Differentially Expressed Genes in Young Panicles of Cas9-OsPAGN1 Lines

**Figure 4:**
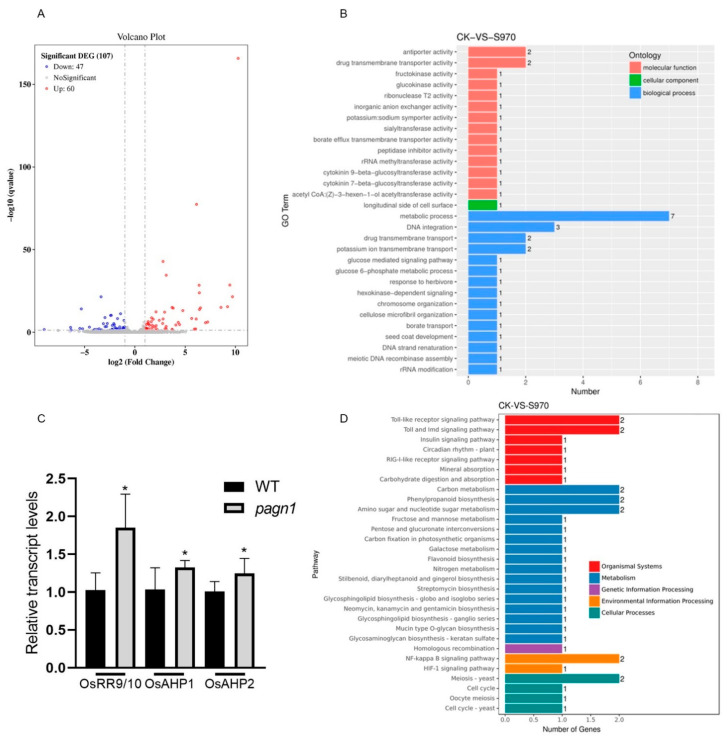
Differentially expressed genes in the young panicles of *OsPAGN1* knockout lines. (**A**) Volcano plot of genes regulated in both *pagn1* plants. (**B**) GO analysis of DEGs. (**C**) Transcript levels of genes involved in the cytokinin signaling pathway. (**D**) KEGG pathway enrichment analysis of DEGs. The data are the mean ± SEM. * *p* < 0.05. (Student’s *t* test).

To identify the DEGs between Wuyugeng and the *OsPAGN1* knockout lines, we performed a transcriptome analysis of the young spikes during the heading period. A volcano plot was constructed for the DEGs (Figure 4A). There were 107 significant DEGs between Wuyugeng and the *OsPAGN1* knockout lines, of which 47 DEGs were up-regulated and 60 DEGs were down-regulated. These DEGs were assigned GO terms (Figure 4B). Most of the DEGs were annotated with biological process GO terms. The most common term was ‘metabolic process’, indicating that *OsPAGN1* broadly affects metabolic activities. In the molecular function category, ‘cytokinin 9-beta-glucosyltransferase activity’ and ‘cytokinin 7-beta-glucosyltransferase activity’ were prominent enriched GO terms. Hence, we conducted a qRT-PCR analysis to examine the expression of cytokinin-related genes. The *OsRR9/10*, *OsAHP1*, and *OsAHP2* expression levels were significantly higher in the *OsPAGN1* knockout plants than in the Wuyugeng plants (Figure 4C).

The DEGs were also analyzed using the KEGG database to identify the enriched pathways (Figure 4D). ‘Carbon metabolism’, ‘phenylpropanoid biosynthesis’, and ‘amino sugar and nucleotide sugar metabolism’ were the main enriched pathways, implying *OsPAGN1* might regulate rice plant architecture through these metabolic processes.

## 3. Discussion

In rice, plant height is a vital agronomic characteristic that directly affects the grain yield [25]. A previous study revealed that the plant biomass increases as the plant height increases, whereas the harvest index remains above 0.5 [1]. The number of tillers and the number of grains per panicle are also among the main factors influencing the rice grain yield [26]. In our study, we observed that *OsPAGN1*, encoding a protein containing a canonical RING motif, positively regulates the plant height, tiller number, and grain number per panicle. In *OsPAGN1* knockout plants, the observed increase in plant height was probably mainly the result of an increase in cell length (Figure 1I,J). We also demonstrated that the RING finger family helps regulate rice plant growth and grain production. A recent investigation suggested that rachis branch formation is a determinant of the number of grains per panicle [27]. The *OsPAGN1* promotor is active in all tissues at the seedling stage as well as in the stamens of young panicles. Additionally, a mutated *OsPAGN1* affects the number of grains per panicle, indicating that *OsPAGN1* might be involved in spikelet primordium formation; however, the underlying molecular mechanism will need to be clarified.

Tomato *fruit weight 2.2* (*fw2.2*) is the first cloned QTL confirmed to substantially contribute to fruit weight and organ size [28,29]. Moreover, *ZmCNR1* (*Zea mays Cell Number Regulator 1*), which is the closest maize ortholog of *fw2.2*, negatively regulates organ size and the overall plant stature. A subsequent study suggested that organ size changes are primarily caused by changes in the number, but not the size, of cells [23]. In rice, *TGW2* encodes CELL NUMBER REGULATOR1 (OsCNR1) and affects grain size and weight by influencing cell proliferation and expansion [30]. In our study, we confirmed that OsPAGN1 can interact directly with OsCNR10, which is similar to OsCNR1, fw2.2, and ZmCNR1 (52%, 50%, and 67% sequence similarities, respectively) (Figure S2). Proteins with homologous sequences usually have similar structures and functions. We speculated that *OsCNR10* may also influence the rice grain yield. We determined that a non-functional *OsCNR10* increases the number of grains per panicle, suggesting *OsCNR10* negatively controls the rice grain yield, which is consistent with the roles of other CNR proteins. Because OsPAGN1 is likely an E3 ubiquitin ligase that interacts with OsCNR10, it may ubiquitinate OsCNR10 to be degraded, thereby regulating the grain yield. The specific relationship between *OsPAGN1* and *OsCNR10* and the underlying mechanism will need to be elucidated in future studies involving genetic and molecular experiments.

Cytokinins have a wide range of functions associated with the regulation of plant growth and development. For example, they regulate cell proliferation by affecting cell division and differentiation [31]. Cytokinin biosynthesis is mediated by a series of enzymes, including adenosine phosphate isopentenyl transferases (IPTs) [32]. Cytokinin histidine kinases HK5 and HK6 function as cytokinin receptors; mutations to both in the *hk5 hk6* double mutant lead to severe dwarfism and stunted rice plants that lack a visible panicle [33]. Downstream of the HKs are two classes of proteins that participate in cytokinin signaling. Authentic His-containing phosphotransfer proteins (HPts) receive a phosphate group from HKs and phosphorylate the response regulators (RRs) [34]. In rice, *OsIPT9* is an important gene for grain filling and determining the rice yield potential [35]. Both OsAHP1 and OsAHP2, which are rice HPts, affect the number of tillers and the seed setting rate in rice [36]. Furthermore, OsRR9 and OsRR10 function as cytokinin signal A-type RRs. The production of A-type RRs is regulated by cytokinins [34,37,38]. The active cytokinin content can be controlled via the irreversible cleavage by cytokinin oxidases. The *Gn1a* (*OsCKX2*) gene encodes a cytokinin oxidase/dehydrogenase, and a decrease in OsCKX2 functionality reportedly enhances rice grain production [39,40]. It can also be modulated by the conjugation involving a sugar [31,41,42,43,44]. The *O-* and *N-*glucosides are the non-active stored forms of cytokinins [45]. Moreover, *O*-glycosylation is catalyzed by cytokinin glucosyltransferases and is reversed by β-glucosidases, whereas *N*-glycosylation is nonreversible [42]. A recent study revealed the potential utility of cytokinin glucosyltransferases for wheat improvement [46]. In our study, we detected differences in the cytokinin glucosyltransferase activity between the wild-type and *OsPAGN1* loss-of-function transgenic plants. We analyzed the expression levels of the cytokinin-related genes mentioned above and revealed the increased expression of *OsAHP1*, *OsAHP2*, and *OsRR9/10*, implying that *OsPAGN1* might be involved in cytokinin signaling pathways and in the maintenance of appropriate levels of active cytokinins in rice.

## 4. Materials and Methods

### 4.1. Plant Materials and Growth Conditions

A *japonica* rice cultivar, Wuyugeng, was used as the wild-type control and for genetic transformations. All plants were grown at the experimental stations of Fudan University in Shanghai, China (31°20′26″ N, 121°30′26″ E) and Hainan (18°18′52″ N, 109°03′05″ E) under natural conditions. The plants were cultivated using standard procedures and field management practices.

### 4.2. Transgene Constructs and Targeted Gene Editing

To generate *OsPAGN1*-overexpressing transgenic plants, the full-length (441 bp) *OsPAGN1* coding sequence (CDS) in Wuyugeng was amplified by PCR and cloned into the pMD19-T vector. The CDS was then inserted into the *pCAMBIA1304* vector to construct the recombinant expression vector (*pCaMV35S::OsPAGN1*). The CRISPR/Cas9 system was used to silence *OsPAGN1* and *OsCNR10* (i.e., gene knockout). A single-guide RNA sequence was inserted downstream of the *OsU6* promoter in the CRISPR/Cas9 binary vector *pBGK032* (Biogle Technology, Jiangsu, China). These recombinant constructs were introduced into embryogenic calli from Wuyugeng via *Agrobacterium tumefaciens*-mediated transformation. Successfully transformed calli were selected by culturing on the selection medium containing 50 mg/L hygromycin for 14 days under dark conditions (28 °C). Drug-resistant calli were selected to transfer into Murashige and Skoog (MS) medium under long-day conditions (16 h: 8 h, light: dark) to grow into plants in the greenhouse. The correctly transformed plants were screened on the basis of PCR amplifications and direct sequencing.

### 4.3. Histological Analysis

Stems at the heading stage were fixed in FAA (100 mL FAA containing 5 mL formalin, 5 mL glacial acetic acid, 5 mL glycerol, and 85 mL 70% ethanol) and then vacuum-infiltrated for 10 min, incubated at room temperature for 16 h, and dehydrated in a gradient series of ethanol solutions. The samples were embedded in Paraplast, sectioned using a microtome, stained with 0.5% Fast Green, and then examined using a microscope (Zeiss, Oberkochen, Germany) and photographed.

### 4.4. β-Glucuronidase (GUS) Staining

To analyze the *OsPAGN1* expression pattern, the promotor fragment (2000-bp region upstream of *OsPAGN1*) was cloned into *pCAMBIA1304* to construct the *OsPAGN1* promoter::GUS reporter gene recombinant vector, which was then used to transform wild-type rice Wuyugeng according to an *A. tumefaciens*-mediated method. Different tissues collected from the transgenic plants were incubated in X-Gluc buffer (1 mg/mL X-Gluc, 10 mM EDTA, 2 mM K_3_[Fe(CN)_6_], 2 mM K_4_[Fe(CN)_6_], 0.02% Triton X-100, 50 mM Na_2_HPO_4_, and 50 mM NaH_2_PO_4_, pH 7.0) at 37 °C for 12 h in darkness. After the GUS staining, the samples were incubated in 75% ethanol to remove chlorophyll before being photographed.

### 4.5. Subcellular Localization

The *OsPAGN1* and *OsCNR10* CDSs were amplified using gene-specific primers (Table S1) and cloned into separate *pYL322-d1* vectors. The resulting recombinant vectors were inserted into rice protoplasts via polyethylene glycol-mediated transformation [47]. Rice protoplasts were grown at 28 °C in darkness for 12–16 h. The fluorescence of the protoplasts was examined using the TCS SP8 confocal laser scanning microscope (Leica, Wetzlar, Germany).

### 4.6. Yeast Two-Hybrid Assay

The *OsPAGN1* and *OsCNR10* CDSs were cloned into *pGBKT7* and *pGADT7*, respectively. The recombinant vectors were inserted into yeast strain Y2HGold cells (Weidibio, Shanghai, China). The transformed cells were grown on synthetic defined medium lacking Leu and Trp (SD/−Leu/−Trp) to select the transformants containing the correct recombinant vector pair or on synthetic defined medium lacking Leu, Trp, His, and Ade (SD/−Leu/−Trp/−His/−Ade) to test for protein interactions.

### 4.7. Split-Luciferase Assay

The *OsPAGN1* and *OsCNR10* CDSs were cloned into the split-luciferase system vectors with sequences encoding nLUC (N-terminal of firefly luciferase) and cLUC (C-terminal of firefly luciferase), respectively. The generated constructs were inserted into *A. tumefaciens* GV3101 cells. The transformed cells were cultured, collected, and resuspended in an infiltration buffer (10 mmol/L MgCl_2_) for a final OD_600_ of 1.0. Equal amounts of the solutions comprising bacterial cells carrying nLUC or cLUC were combined and injected into 4-week-old tobacco (*Nicotiana benthamiana*) leaves. Three days later, the luciferase luminescence signals were detected using the NightShade LB 985 in vivo plant imaging system.

### 4.8. GST Pull-Down Assay

The *OsPAGN1* CDS was inserted into *BamH*I and *EcoR*I sites of the *pGEX-4T-1* vector and the *OsCNR10* CDS was inserted into *Nde*I and *Sal*I sites of the *pCold-TF* vector for the subsequent production of GST-tagged OsPAGN1 and poly-His-tagged OsCNR10. The fusion proteins and tags were expressed in *Escherichia coli* BL21 (DE3) cells. Isopropyl-β-D-thiogalactopyranoside (final concentration of 0.5 mM) was added when the OD_600_ = 0.6 to induce the expression of mentioned constructs at 15 °C for 16 h. The GST-OsPAGN1 fusion protein or GST alone was added to PBS buffer (10 mM phosphate, 150 mM NaCl, 0.5 mM PMSF, 0.1% Triton, and 0.5 mM EDTA, pH 7.2) containing an equal amount of His-OsCNR10 and 50 μL GST beads in 1.5-mL centrifuge tubes. After an overnight incubation at 4 °C with gentle mixing (i.e., on a rotator), the beads were washed three times with PBS buffer and boiled at 95 °C for 5 min. The proteins were separated by 10% sodium dodecyl sulfate–polyacrylamide gel electrophoresis and then analyzed using anti-GST or anti-His antibodies (GNI, Tokyo, Japan).

### 4.9. RNA-Seq Analysis

Total RNA was extracted from the young spikes (i.e., at the booting stage) of Wuyugeng and *OsPAGN1* knockout plants using the FastPure Plant Total RNA Isolation Kit (Vazyme, Nanjing, China). The extracted RNA was used as the template for constructing cDNA libraries according to Illumina standard protocols. The libraries were sequenced using the Illumina HiSeq 4000 platform by GENEWIZ (Suzhou, China). Gene expression levels were calculated and recorded in terms of fragments per kilobase of transcript per million mapped reads (FPKM) values. Differentially expressed genes (DEGs) were automatically annotated and manually categorized according to their putative or demonstrated function. More specifically, GOSeq (v1.34.1) was used to identify Gene Ontology (GO) terms that annotate a list of enriched genes with a significant padj less than 0.05. Additionally, topGO was used to plot DAG. KEGG (Kyoto Encyclopedia of Genes and Genomes) is a collection of databases dealing with genomes, biological pathways, diseases, drugs, and chemical substances (http://en.wikipedia.org/wiki/KEGG (accessed on 10 December 2021)). We used scripts in house to enrich significant differential expression genes in KEGG pathways.

### 4.10. qRT-PCR Analysis

Total RNA was extracted using the FastPure Plant Total RNA Isolation Kit (Vazyme, Nanjing, Jiangsu, China). First-strand cDNA was synthesized using HiScript III All-in-one RT SuperMix Perfect for qPCR (Vazyme, Nanjing, China). The qRT-PCR analysis was performed using TB Green Premix Ex Taq II (Takara Biomedical Technology, Beijing, China). Relative gene expression levels were calculated using a ubiquitin-encoding gene as an internal reference control. The qRT-PCR primers are listed in Table S4.

## Data Availability

Sequence data from this article are available at the Rice Genome Annotation Project, http://rice.uga.edu/ (accessed on 10 December 2021) (accession nos. LOC_Os07g23970 [*OsPAGN1*] and LOC_Os03g61470 [*OsCNR10*]), both cDNAs were cloned from Wuyugeng.

## Supplementary Materials

The following are available online at https://www.mdpi.com/article/10.3390/ijms23020824/s1.
