# Supplementary material for: A New RING Finger Protein, PLANT ARCHITECTURE and GRAIN NUMBER 1, Affects Plant Architecture and Grain Yield in Rice
Source: Int J Mol Sci. 2022 Jan 13;23(2):824. doi: 10.3390/ijms23020824 (PMC8777624; doi:10.3390/ijms23020824)
Supplement: Supplementary file 1 [file ijms-23-00824-s001.zip › ijms-1526387-supplementary.pdf]

Supporting Information

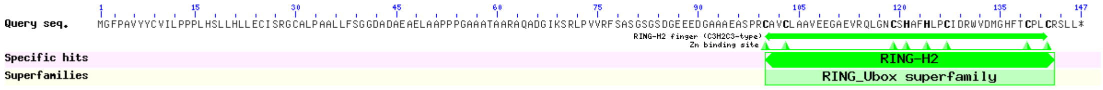

FigureS1. Conserved domain of OsPAGN1.

```
OsCNR1      -MYPSAPPDAYNKYSAGAPPTAPP---PATYQLPTMNTPRTGGGLTRWSTGLFHCMDDPG
fw2.2       -MYQTV---GYNPGPMKQPY------------VPPHYVSAPGTTTARWSTGLCHCFDDPA
OsCNR10     MAKPSAAP------VTGVPV-----------------------GSAAWSTGLCDCFDDCG
ZmCNR2      -MYPKAADEGAQPLATGIPFSGGGGYYQAGGAMAAAFAVQAQAPVAAWSTGLCNCFDDCH
               ..            *                           : *****  *:**

OsCNR1      NCLITCVCPCITFGQVADIVDKGTCPCLASGTAYALLCA-SGMGCLYSCFYRSKMRAQFD
fw2.2       NCLVTSVCPCITFGQISEILNKGTTSCGSRGALYCLLGL-TGLPSLYSCFYRSKMRGQYD
OsCNR10     LCCLTCWCPCITFGRVAEMVDRGSTSCGTGGALYGLLCAFTGCQWIYSCTYRGKMRTQYG
ZmCNR2      NCCVTCVCPCITFGQTAEIIDRGSTSCGTSGALYALVMLLTGCQCVYSCFYRAKMRAQYG
             * :*. *******. :::::.*: .* : *: * *:    :*    :*** **.*** *:.

OsCNR1      LDEGDCPDFLVHFCCEYCALCQEYRELKNRGFDLGIGWAANVDRQRRGVTGASVMGAPGV
fw2.2       LEEAPCVDCLVHVFCEPCALCQEYRELKNRGFDMGIGWQANMDRQSRGVT----------
OsCNR10     LAEAGCADCCVHFCCEPCALCQEYRELVARGYDPKLGWHLNADRAAAAGA----------
ZmCNR2      LQVSPCSDCCVHCCCQCCALCQEYRELKKRGFDMSIGWHANMERQGRAAA----------
            *  . * *  **  *: **********  **:*  :**  * :*    . :

OsCNR1      PVGMMRAAPAVQY--MGR
fw2.2       -------MPPY-HAGMTR
OsCNR10     -------APAVQY--MGR
ZmCNR2      ------AVPPHMHPGMTR
                   *.   :   * *
```

Figure S2 Alignment between OsCNR1, fw2.2, OsCNR10 and ZmCNR10.

Table S1. RING-finger family genes highly expressed in seedling and panicles

Gene NO.	seedling-TRAP-Seq FPKM	panicle-TRAP-Seq FPKM
LOC_Os07g23970(*OsPAGN1*)	2.14486	2.49139
LOC_Os02g32570	0.442666	8.82987
LOC_Os03g05270	3.98811	5.07989
LOC_Os12g05370	1.55616	5.52752
LOC_Os07g32730	0.569401	8.49283

Table S2. Conservation of OsPAGN1's conserved domain amino acid sequences among various plant species

Entry	Organism	E-value	Score	Identity
A0A0E0LJT2	*Oryza punctata*	8.60E-35	307	100.0%
B8B5I0	*Oryza sativa* subsp. *indica*	1.10E-34	307	100.0%
A0A0E0IPG5	*Oryza nivara*	1.10E-34	307	100.0%
A0A0E0Q7B6	*Oryza rufipogon*	1.10E-34	307	100.0%
A0A0E0EAH4	*Oryza meridionalis*	1.20E-34	307	100.0%
A0A0D3GQ77	*Oryza barthii*	1.20E-34	307	100.0%
A0A6G1D702	*Oryza meyeriana* var. *granulata*	1.40E-34	303	97.7%
A0A0E0AIX5	*Oryza glumipatula*	1.00E-33	301	97.7%
A0A0D9WY60	*Leersia perrieri*	3.70E-33	297	95.5%
J3MKA5	*Oryza brachyantha*	4.10E-31	283	90.9%
A0A4S8JEG7	*Musa balbisiana*	2.20E-21	221	77.3%
A0A2I0AXB9	*Apostasia shenzhenica Ananas*	5.30E-21	219	70.5%
A0A199W6Y1	*comosus*	7.70E-19	203	70.5%
A0A199V923	*Ananas comosus*	8.20E-19	203	70.5%
C4J4V7	*Zea mays*	1.40E-18	202	65.9%
B4FM70	*Zea mays*	1.50E-18	202	65.9%
A0A3L6RSA2	*Panicum miliaceum*	3.10E-18	200	68.2%
C5Y0X8	*Sorghum bicolor*	4.60E-18	199	65.9%
K3Y990	*Setaria italica*	5.00E-18	199	68.2%

Table S3. Conservation of Os970 amino acid sequences among rice

Entry	Organism	E-value	Score	Identity
B8B5I0	*Oryza sativa* subsp. *indica*	1.30E-104	775	100.0%
A0A0E0IPG5	*Oryza nivara*	1.30E-104	775	100.0%
A0A0E0Q7B6	*Oryza rufipogon*	7.30E-104	770	99.3%
A0A0E0AIX5	*Oryza glumipatula*	4.90E-96	719	92.1%
A0A0D3GQ77	*Oryza barthii*	4.90E-96	719	92.1%
A0A0E0EAH4	*Oryza meridionalis*	1.30E-93	703	90.7%
A0A0E0LJT2	*Oryza punctata*	2.40E-83	635	84.2%
A0A0D9WY60	*Leersia perrieri*	1.10E-73	572	75.2%
J3MKA5	*Oryza brachyantha*	1.80E-68	537	73.6%
A0A6G1D702	*Oryza meyeriana* var. *granulata*	1.70E-65	516	74.1%

Table S4. Primers used in this study

Primer Name	Primer Sequence
**Primers for q-PCR**	
q-OsIPT9-F	ACACCCGAACAACCATCGAA
q-OsIPT9-R	GTCGACCCCACTTCTCTGTG
q-OsIPT10-F	CTGCCTTCTCCTTGCCCTAC
q-OsIPT10-R	CAGCATCTCTTCGCACCTCA
q-LOG-F	CGGTTTGCTGATGCGTTCAT
q-LOG-R	TCGCTGACAGCCATGTCAAT
q-OsCKX4-F	GACCGACTACCTCCATCTCACA
q-OsCKX4-R	GGTTGACATTGCTGACCTGC
q-OsRR9/10-F	TCATGAGGACAGCCCAATTTCTA
q-OsRR9/10-R	TGCAGTAGTCTGTGATGATCAGGTT
q-OHK4-F	CAAGAACATATTGGTAGTTG
q-OHK4-R	CCTTCCCTTCCATTGCTCGT
UBQ5-F	AGCAGAAGCACAAGCACAA
UBQ5-R	AAGCCTGCTGGTTGTAGACG
**Primers for subcellular localization**	
GFPN-CNR10-F	CCATTTACGAACGATAGCCATGGCCAAGCCAAGCGCCGCT
GFPN-CNR10-R	ccttgctcaccatCAGGATCCCGCGGCCCATGTACTGCAC
GFPN- PAGN1-F	CCATTTACGAACGATAGCCATGGCTTCCCGGCGGTGTAC
GFPN- PAGN1-R	ccttgctcaccatCAGGATCCCGAGGAGAGATCTGCAGAG
**Primers for construct *pPAGN1:GUS***	
GUS-PAGN1-F	GCCAAGCTTGCATGCCTGCAGagtattttgatttctaaaa
GUS-PAGN1-R	GGACTGACCACCCGGGGATCCgggtttgtatggtggaaaa
**Primers for expression construct**	
BD-PAGN1-F	TCAGAGGAGGACCTGCATATGATGGGCTTCCCGGCGGTGT
BD-PAGN1-R	GCAGGTCGACGGATCCCCGGGTCAGAGGAGAGATCTGCAG
AD-CNR-F	CGTACCAGATTACGCTCATATGATGGCCAAGCCAAGCGCC
AD-CNR-R	GATGCCCACCCGGGTGGAATTCTTAGCGGCCCATGTACTG
771-PAGN1-F	cacgggggacgagctcggtaccATGGGCTTCCCGGCGGTG
771-PAGN1-R	acgcgtacgagatctggtcgacGAGGAGAGATCTGCAGAG
772-CNR10-F	gtacgcgtcccggggcggtaccATGGCCAAGCCAAGCGCC
772-CNR10-R	aacgaaagctctgcaggtcgacTTAGCGGCCCATGTACTG
PGEX4T-PAGN1-F	GGATCTGGTTCCGCGTGGATCCATGGGCTTCCCGGCGGTG
PGEX4T-PAGN1-R	GCTCGAGTCGACCCGGGAATTCTCAGAGGAGAGATCTGCA
pCold-CNR10-F	TGGTATCGAAGGTAGGCATATGATGGCCAAGCCAAGCGCC
pCold-CNR10-R	CCTATCTAGACTGCAGGTCGACTTAGCGGCCCATGTACTG
p104-PAGN1-F	TTACAATTACAGGTACCCGGGATGGGCTTCCCGGCGGTG
p104-PAGN1-R	CACGCTGCCACCGCCGTCGACGAGGAGAGATCTGCAGAG
p106-CNR10-F	ATCGAGGACGCCGGCGGATCCATGGCCAAGCCAAGCGCC
p106-CNR10-R	ACGAAAGCTCTGCAGGTCGACTTAGCGGCCCATGTACTG
GFPN-CNR10-F	CCATTTACGAACGATAGCCATGGCCAAGCCAAGCGCCGCT
GFPN-CNR10-R	ccttgctcaccatCAGGATCCCGCGGCCCATGTACTGCAC
GFPN-PAGN1-F	CCATTTACGAACGATAGCCATGGCTTCCCGGCGGTGTAC
GFPN-PAGN1-R	ccttgctcaccatCAGGATCCCGAGGAGAGATCTGCAGAG
